# Ischemic Heart Disease during Acute Exacerbations of COPD

**DOI:** 10.3390/medsci6040083

**Published:** 2018-09-25

**Authors:** Rosa Malo de Molina, Silvia Aguado, Carlos Arellano, Manuel Valle, Piedad Ussetti

**Affiliations:** 1 Department of Pulmonary Medicine, University Hospital Puerta de Hierro, Majadahonda, 28040 Madrid, Spain; s.aguado.ibanez@gmail.com (S.A.); rycmvalle@hotmail.com (M.V.); pied2152@separ.es (P.U.); 2 Department of Cardiology, University Hospital Puerta de Hierro, Majadahonda, 28040 Madrid, Spain; arellano.serrano@gmail.com

**Keywords:** chronic obstructive pulmonary disease, cardiovascular, myocardial infarction, exacerbation, treatment, beta-blockers

## Abstract

Patients with chronic obstructive pulmonary disease (COPD) have a higher risk of acute cardiovascular events, and around 30% die from cardiovascular diseases. Recent data suggest an increased risk of myocardial infarction in the following days of a severe exacerbation of COPD. Disruption in the balance during the exacerbation with tachycardia, increased inflammation and systemic oxidative stress as well as some other factors may confer an increased risk of subsequent cardiovascular events. A number of investigations may be useful to an early diagnosis, including electrocardiography, imaging techniques and blood test for biomarkers. Some drugs that have changed prognosis in the cardiovascular setting such as cardioselective beta-blockers may be underused in patients with COPD despite its demonstrated benefits. This review focuses on several aspects of exacerbation of COPD and cardiovascular events including epidemiology, possible mechanism, diagnosis and treatment.

## 1. Introduction

Patients with chronic obstructive pulmonary disease (COPD) often develop cardiovascular comorbidities [1]. In addition, they have a higher risk of acute cardiovascular events, and around 30% die from cardiovascular diseases (CVD) [2,3].

Focusing on ischemic heart disease, a recent meta-analysis showed that patients with COPD had a consistent risks of coronary heart disease (meta-odd ratio (OR) 186, 95% confidence interval (CI) 151–230; *p* < 0.0001), myocardial infarction (271, 169–435; *p* < 0.0001), and angina pectoris (816, 308–2159; *p* <0.0001) [4].

Indeed, COPD patients with acute ischemic heart diseases (IHD) may have worse outcomes. The three-year follow-up of 4284 patients who received hospital treatment for coronary heart disease reported mortality rates of 21% for patients diagnosed with COPD versus 9% in those without COPD (*p* ≤ 0.001) [5].

COPD patients who develop ST-segment elevation myocardial infarction (STEMI) at three-year follow-up had higher mortality than non-COPD patients in a web-based Italian registry of 11,118 consecutive patients with STEMI.

Remarkably, hospital readmissions for COPD emerged as a strong independent risk factor for recurrence of MI (HR, 2.1; 95% CI, 1.4–3.3) [6].

It is likely that part of this increased risk of IHD comes from shared risk factors, such as smoking or sedentary behavior, but also the increased cardiac stress associated with acute exacerbations that COPD patients suffer may play an important role [2].

In this line of research, recent data from the UK suggest a 2.58-fold increased risk of myocardial infarction (MI) in the following 91 days of a severe acute exacerbation of COPD (AECOPD) [7]. Notably, the cross-sectional nature of the evidence confers difficulties in assessing the directions of the association: whether myocardial infarction contributes to AECOPD or vice versa. Although the prevalence of myocardial infarction following hospitalization for AECOPD has been broadly described, no data related to the prevalence of COPD exacerbations during acute coronary events have been published to date.

The purpose of this review is to summarize current knowledge relating to epidemiology, diagnosis and treatment of ischemic heart disease in people with AECOPD and the mechanisms that may underlie its coexistence.

A search was conducted in MEDLINE (via PubMed) for observational studies published between January 1990 and August 2018 reporting ischemic heart disease including coronary heart disease, myocardial infarction, and angina pectoris in patients with acute exacerbations of COPD.

## 2. Epidemiology

Myocardial infarction is frequently unrecognized in patients hospitalized for COPD exacerbation. Five main articles analyzed the association between AECOPD and acute coronary events (Table 1). Brekke and coauthors retrospectively analyzed the electrocardiogram (ECG) from the day of admission of 897 patients with at least one AECOPD hospitalization. Of the 229 (25%) patients with ECG signs of previous infarction, only 30% (CI 24–36%, *n* = 68) had a recognized history of MI [8]. Unfortunately, they used a Cardiac Infarction Injury Score (CIIS) which does not include ST-segment changes in the algorithm and only 84% of COPD patients included had a spirometry in their medical record [8].

Donalson et al., using UK administrative data from 25,857 patients with COPD, reported an incidence rate of MI of 1.1 per 100 patient-years with a 2.27-fold (95% CI 1.1–4.7; *p* = 0.03) increased risk of MI 1–5 days after exacerbation (defined by prescription of both steroids and antibiotics) [9].

They analyzed aself-controlled case series of COPD, avoiding the need for multivariate analysis to adjust for confounders such as socioeconomic background or family history. However, the study has some limitations due to its retrospective nature. The study used administrative codes for COPD and MI and only some COPD patient had spirometry data recorded. Indeed, the date of the exacerbation onset could not be determined precisely only by the date of consultation and this may overestimate the time window for an acute coronary event.

Some of the limitations were solved by a prospective case series study looking at the incidence of MI in patients hospitalized with an acute exacerbation of COPD (*n* =242) in four hospitals. In total, 124 COPD patients (51%; 95% CI 48–58%) developed chest pain during the exacerbation, and 24 (10%) had raised troponin (Tn), among whom 20 (8.3%; 95% CI 5.1–12.5%) had chest pain and/or serial ECG changes, fulfilling the 2007 Universal Definition of Myocardial Infarction [10].

One in 12 patients had raised Tn along with serial ECG changes and/or chest pain and therefore a myocardial infarction diagnosis [10]. Patients with chest pain at admission were excluded.

From our point of view, patients whose primary presenting complaint was chest pain and have a diagnosis of AECOPD during hospital admission should also be evaluated, as being hospitalized by a MI does not exclude having symptoms concordant with an AECOPD before arrivingto hospital.

Recent data from the SUMMIT trial (Study to Understand Mortality and MorbidITy), which investigated the effects of vilanterol and fluticasone furoate, by means of a secondary prospective cohort analysis of COPD patients with cardiovascular disease or cardiovascular risk, proved that exacerbations conferred an increased risk of subsequent CVD events, especially in hospitalized patients and within the first 30 days post-exacerbation. CVD events were a composite outcome of cardiovascular death, myocardial infarction, stroke, unstable angina, and transient ischemic attack. Among 16,485 participants in SUMMIT, 4704 participants had at least one AECOPD, 688 had at least one CVD event and 173 myocardial infarction. The 30-day hazard ratio (HR) for CV events following hospitalized AECOPD was more than two-fold higher than non-hospitalized COPD exacerbations [11].

Analysis restricted to only myocardial infarction events showed similar results, with a substantially increased risk of myocardial infarction in the first 30 days following AECOPD, a lower, but still significant, risk between 31days and 1 year, and no significant increased risk beyond 1 year. These data do not apply to COPD patients without CVD or CVD risk factor or those with severe airflow limitation (forced expiratory volume in 1 second or FEV 1 < 50%) [11].

Lately, in a within-individual analysis of 5696 COPD patients, from the UK Clinical Practice Research Datalink with linked Hospital Episodes Statistics data, Rothnie et al., investigated the rates of myocardial infarction and ischemic stroke following acute exacerbation compared to stable time [7]. They included 2850 COPD patients with a first myocardial infarction and at least one acute exacerbation, defined by a using prescription of antibiotics and oral steroids. Compared to stable periods, the 91 days following the onset of acute exacerbation were associated with a 65% increased risk of myocardial infarction (IRR 1.65, 95% CI 1.50–1.81). The increased myocardial infarction risk peaked in the first threedays post-AECOPD. The risk gradually fell back to a stable period level after 28 days for myocardial infarction.

The strength of this study was the sample size and also the analysisof previous cardiovascular medications, COPD medication, severity of airflow limitation and severity of AECOPD defined by those requiring hospitalization. However, the results in term of covariables were somewhat surprising as there was no modification of the associations between acute exacerbation and myocardial infarction by other previous cardiovascular diseases, cardiovascular drugs, COPD medicines, influenza vaccine in the baseline period, or by age or sex.

However, it is worth noting that those patients in whom primary prevention with these medicines was completely effective would not have been included in the study due to the case nature of the design.

More expected was the stronger association of acute exacerbation with myocardial infarction for those with severe airflow limitationas compared to mild-to-moderate (global initiative for chronic obstructive lung disease (GOLD) grade 1–2 IRR 1.69, (95% CI 1.45–1.98); GOLD grade 3–4 IRR 1.98, (95% CI 1.61–2.05); *p* = 0.007). With the evidence that we have now, mostly cross-sectional, it is not possible to assess the directions of the association (i.e., whether cardiovascular disease contributes to COPD or vice versa).

## 3. Possible Mechanisms of Ischemic Heart Diseases during Acute Exacerbations of COPD.

**Figure 1:**
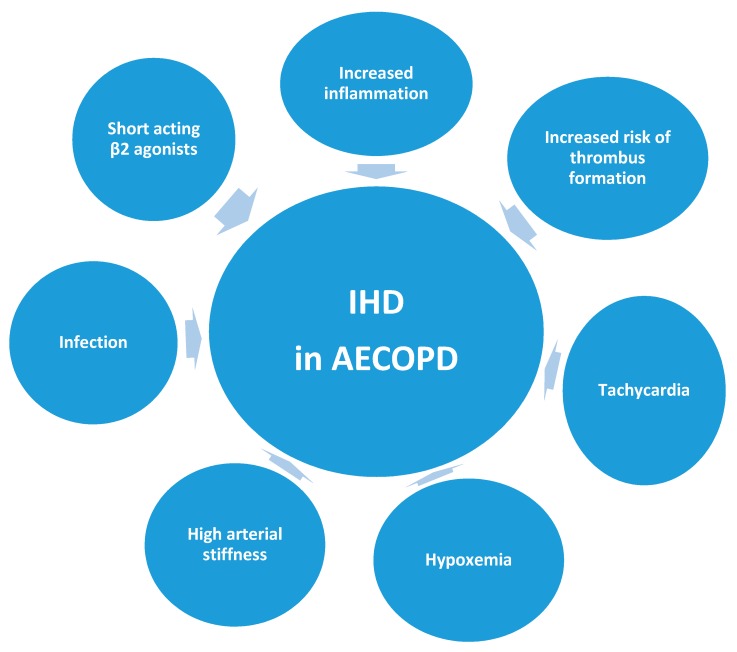
Potential factors contributing to acute ischemic heart disease in exacerbations of COPD. IHD: ischemic heart disease.

In this section, we briefly explore some of the mechanisms that are believed to underlie the association between AECOPD and IHD (Figure 1).

### 3.1. Infection as the Causal Mechanism of Acute Exacerbation of COPD (AECOPD)

Studies have shown that respiratory infection in the general population increases the likelihood of a MI. Meier et al. [12] reported a relative risk of 2.7 in healthy individuals during Days 1–5. Smeeth et al. [13], who investigated the relationship between lower respiratory tract infection (LRTI) and risks of myocardial infarction in 20,921 people from the general population, also reported a relative risk of 4.95 (95% CI 4.43–5.53) for myocardial infarction over Days 1–3 following LRTI. Clayton et al. [14] similarly reported in a case-controlled study an odds ratio of 2.10 with recent respiratory infection.

Whether this increased risk of MI in patients with AECOPD is restricted to those with infection as the causal mechanism of the exacerbation needs to be elucidated.

### 3.2. Increased Inflammation

Most COPD exacerbations are due to lower respiratory tract infections that are associated with an acute phase response with a rise in systemic inflammatory markers such as C reactive protein (CRP) [15].

This inflammatory state has been postulated as a causal mechanism of the increased risk of IHD during AECOPD.

Interestingly, systemic inflammatory mediators in stable COPD (such as CRP, fibrinogen, surfactant protein D, and neutrophils) have been associated with increased risk of CVD morbidity and mortality [16,17,18].

However, although previous studies suggested a potent independent association of CRP levels with cardiac events, the strength of this association has been shown to be weaker than previously reported in a recent large meta-analysis and in prospective studies [19].

Vanhaverbeke and colleagues, prospectively measured CRP in patients with a MI admitted to hospital. Median CRP levels were 1.89mg/L on admission with MI and peaked to 12.10 mg/L during hospitalization [20]. It seems that the inflammatory state was not the causal mechanism and the posterior inflammatory response is secondary to myocardial repair. Unfortunately, those were not COPD patients, but it would be interesting to perform a prospective measurement of this biomarker in a cohort of patients with AECOPD and MI and see if CRP may have different implications.

None of the observational studies mentioned in the epidemiology section of the review measured PCR. For this reason, to date, it is not possible to determine its potential contribution of inflammation in AECOPD to acute coronary events.

### 3.3. Increased Risk of Thrombus Formation

Platelet activation is present in stable COPD and increases during exacerbations [21]. Concentrations of other pro-atherothrombotic biomarkers, such as interleukins 6 and 8 and tumor necrosis factor α, and prothrombin fragments are also raised during exacerbations. These findings suggest that exacerbations of COPD lead to endothelial dysfunction and precipitate atherosclerotic plaque rupture and thrombosis [22].

Another possible explanation would be the increase in fibrinogen during AECOPD [23], biomarker that is directly thrombogenic [24].

### 3.4. Tachycardia

Hypoxemia and tachycardia associated with a COPD exacerbation can lead to adverse coronary artery events, especially in patients with pre-existing CHD or left ventricular dysfunction. The association between elevated heart rate and progression of coronary atherosclerosis has been shown in clinical studies. High minimum heart rate measured during a 24-h period in young men who survived a first myocardial infarction was associated with progression of both diffuse lesions and distinct stenos measured by angiography [25].

A positive association between a mean heart rate >80 bpm and the development of plaque disruption measured by coronary angiogram was found in an age- and sex-matched case-control study [26].

Indeed, some evidence shows that the mortality associated with increased troponin concentrationsin patients with COPD exacerbations might be linked to tachycardia [27]. This necessitates paying attention to cardiac frequency an adequately controlling it in case of AECOPD.

### 3.5. High Arterial Stiffness

Carotid-femoral aortic pulse wave velocity (aPWV) is a repeatable, validated gold standard noninvasive marker of cardiovascular risk and mortality, in apparently healthy and disease-specific populations. Increments of 1 ms^−1^ are associated with an increase in cardiovascular events and mortality of 12–18% depending on the risk profile of the population studied [28]. Higher arterial stiffness increases myocardial work against elevated systolic aortic pressures and reduces diastolic coronary artery blood flow, which in health is augmented by a slow reflected pulse wave arriving back at the coronary vasculature during diastole [29].

COPD exacerbations also provoke acute increases in arterial stiffness (assessed by aPWV) which then increases left ventricular afterload [30].

Arterial stiffness rose an average of 1.2 ms^−1^ (11.1%) from stable state to exacerbation (*n* = 55) and fell slowly during recovery in a prospective study of 98 COPD patients. In those with airway infection at exacerbation (*n* = 24), this rise was greater (1.4 ± 1.6 vs. 0.7 ± 1.3ms^−1^; *p* < 0.048), prolonged, and related to sputum interleukin-6 (IL-6, rho < 0.753; *p* = 0, 0.001). In addition, the fact that exacerbation frequency status is independently associated with arterial stiffness raises the important possibility that acute exacerbations may accelerate this chronic ongoing cardiovascular process [30].

### 3.6. Short Acting β2 Agonists

High-dose of short acting β2 agonist inhaler therapy used to treat acute exacerbations of COPD might affect the β1 pathway and further aggravate tachycardia and sympathetic stress. These drugs are considered safe at standard doses in patients with stable COPD. However, the safety of these high doses during exacerbations has not been established in clinical trials. Although there is no evidence to confirm the hypothesis in patients with acute exacerbations of COPD at risk of ischemic heart diseases, high doses of β2 agonist may predispose to acute coronary events.

### 3.7. Hypoxemia

AECOPD may be associated with acute hypoxemia. It has been demonstrated that acute hypoxemia increases sympathetic nerve activity by stimulation of arterial chemoreceptors [31]. This sympathetic drive may link tachycardia and increases risk of ischemic coronary events [25,26]. 

## 4. Diagnosis

The diagnosis of heart disease in patients with COPD exacerbation is difficult, because many signs and symptoms are overlapping. The objective is to recognize acute coronary artery events in AECOPD as soon as possible. A delayed diagnosis of myocardial infarction in COPD patients has been demonstrated.In a UK database, 34,027 COPD patients with a first diagnosis of ST-elevation myocardial infarction (STEMI) were compared to 266,119 non-COPD patients. Patients with COPD who had a STEMI were more likely to have an initial diagnosis other than definite STEMI (OR 1.24, 95% CI 1.19–1.30) [32]. The following subsections discuss various diagnostic techniques.

### 4.1. Electrocardiogram

Acute exacerbated COPD patients should be evaluated for signs of acute ischemia in ECG (ST-segment depression or elevation), and previous MI (T-wave inversion, pathological Q-wave, loss of R, or left bundle branch block). In the 2007 Universal Definition statement, myocardial infarction was defined as a rise and/or fall of Tn concentration together with evidence of myocardial ischemia (at least one of the following: symptoms of ischemia; new ST-T changes; new left bundle branch block; or development of pathological Q waves in the ECG) [33].

McAllister and coauthors studied patients hospitalized with AECOPD and found that ECG was frequent abnormal: 12 (5%) had left bundle branch block, 34 (14%) T wave inversion, and 37 (15%) Q waves considered “diagnostic” in the Minnesota coding system. Only three patients developed Q waves during admission, but serial changes in T wave inversion/flattening and ST depression were common (65 (32%) and 19 (9%), respectively). Thirteen(6%) had serial changes in ST elevation. These changes in the T wave axis and dynamic ST segment depression on serial ECG, although common, were not associated with raised troponin [10]. These changes may reflect transient myocardial ischemia secondary to increased oxygen demand or reduced supply that is insufficient to induce MI.

Rothnie et al., in a self-controlled case-series of 5696 adults with COPD with a first myocardial infarction (*n* = 2850), investigated the rates of myocardial infarction following acute exacerbation compared to stable time, within individuals, and found that the association of acute exacerbation with myocardial infarction in the 91 days following acute exacerbation was higher for non-STEMIs (IRR 1.80, 95% CI 1.56–2.06) than for STEMIs (IRR 1.39, 95% CI 1.16–1.68) [7]. From a clinical point of view, these findings may help us to carefully look for these kinds of ECG alterations in AECOPD.

### 4.2. Echocardiogram

An echocardiogram may assess biventricular cardiac function and identify ischemic areas when IHD is suspected. However, it is often less useful in an exacerbating patient as a high proportion of tests are limited by poor acoustic window.

No data related to echocardiography findings in exacerbated COPD patients with acute ischemic heart disease have been published yet.

### 4.3. Cardiac Magnetic Resonance

Currently, the gold standard for assessing biventricular function, cardiac morphology and viability is Cardiac MRI, especially in patients with poor acoustic window on echocardiogram. However, this technique has important limitations: long duration, cost and claustrophobic nature of an MRI scanner. These limitations make this technique impractical in patients with COPD exacerbation [34].

### 4.4. Cardiac Computed Tomography (CT)

In patients with suspected coronary artery disease, multicenter studies using 64-slice CT have demonstrated sensitivities of 95–99% and specificities of 64–83% as well as negative predictive values of 97–99% for the identification of individuals with at least one coronary artery stenosis [35].

Dynamic cardiac CT can be used to assess biventricular function, pulmonary artery anatomy, coronary artery calcification, and pulmonary structure. In exacerbated COPD patient, this technique may also be a good diagnostic strategy.

Interestingly, low-dose ungated multidetector CT performed for lung evaluations is reliable to predict the presence of coronary artery calcification (CAC) and assessment of Agatston score. Correlations between gated and ungated CAC were excellent (*r* = 0.96) [36].

### 4.5. Biomarkers

Both troponin (Tn) and brain natriuretic peptide (BNP) are markers of myocardial stress, which can be easily measured and relatively cheap as a bedside test.

A rise of Tn concentration is a necessary condition to diagnose myocardial infarction [33]. However, although Tn measurements are specific for myocardial necrosis, they are not specific for ischemic injury because cardiac Tn can also be raised in heart failure, renal dysfunction, pulmonary embolism, pulmonary hypertension, tachyarrhythmias, and sepsis.

In most cases, during exacerbations, high sensitivity measurements show higher circulating cardiac Tn concentrations than the upper normal limit, particularly in patients with known ischemic heart disease [27,37,38].

In a meta-analysis of eight studies of patients with AECOPD, Cardiac Tn elevation ranged from 18% to 73%. They found that cardiac Tn elevation was significantly related to an increased risk for all-cause mortality (OR 1.69; 95% CI 1.25–2.29; I2 40%). This finding was independent to the follow-up length of studies (≤6 months: OR 3.22, 95% CI 1.31–7.91; >6 months: OR 1.38,95% CI 1.02–1.86). When they compared the kind of troponin with the mortality prediction, Tn T seemed to be more helpful in predicting all-cause mortality as compared to Tn I (OR 1.54,95% CI 1.2–1.96 vs. OR 3.39, 95% CI 0.86–13.36, respectively) [37].

In a recent issue of Open Heart, Buhan et al. reviewed the role of cardiac biomarkers for predicting left ventricular dysfunction and cardiovascular mortality in acute exacerbations of COPD [39].

Of the ten included papers that measured Tn, seven analyzed mortality, and, in all of these, a significant association was found between elevated levels of Tn and increased mortality. Most of the studies were generally long term (more than 30 days after measurement), although various time points were used in the follow up (from in-hospital deaths to 50 months). In addition, increased levels of Tn at discharge was a predictor of readmission to hospital (HR = 2.89, 95% CI 1.13–7.36) [39]. In addition, not only the Tn level but also whether it rises, then falls or remains elevated during an AECOPD may also have prognostic value [40].

Brain natriuretic peptide is not useful to predict acute ischemic heart disease but may be important to assess left ventricular failure that may accompany MI in some exacerbated COPD patients. Another important reason to measure BNP or NT-proBNP in AECOPD is because its raise is associated to increased short term mortality. The measure of NT-proBNP during AECOPD then is justified as a prognosis marker and as a strategy to diagnose cardiac failure [39].

## 5. Treatment

Patients with AECOPD who develop MI should be treated as guidelines and no restriction should be made with cardiovascular medication. It has been shown that patients with COPD receive less guideline-recommended treatment for CVD, such as revascularization, than patients with CVD but without COPD [32].

We summarizesome data on cardiovascular treatment and AECOPD.

### 5.1. Acetylic Salicylic Acid (ASA)

In a prospective observationalstudy of 1343 patients with COPD who were admitted to hospital with acute exacerbation [35], thrombocytosis (>400 × 10⁹ cells per mm^3^) at admission was independently associated with increased in-hospital and one-year all-cause mortality, and antiplatelet therapy was associated with reduced one-year all-cause mortality after an acute exacerbation of COPD [41].

This has been verified in subsequent meta-analysis [42] in which all-cause mortality was significantly lower in COPD patients receiving antiplatelet treatment (OR 0.81,95%CI 0.75–0.88). This association was observed inboth stable COPD patients and those with acute exacerbation of COPD. Overall, the five studies included 11,117 COPD patients, 3069 with AECOPD. Antiplatelet therapy administration was common (47%, 95% CI 46–48%), ranging from 26% to 61%. A strength of the studies included was that IHD, present in 33% of COPD patients, was analyzed as a confounding factor by all the authors.

In this line, a recent retrospective cohort study using a large population-based data of 206,686 patients hospitalized for AECOPD, aspirin use was associated with lower rates of in-hospital mortality (1.0% vs. 1.4%; OR 0.60 (95% CI 0.50–0.72); *p*  < 0.001), invasive mechanical ventilation use (1.7% vs. 2.6%; OR 0.64 (95% CI 0.55–0.73); *p* < 0.001), and shorter length-of-stay compared to non-users [43].

In a recent self-control case series, there was no modification of the increased risk of myocardial infarction in acute exacerbation compared to stable stateby previous aspirin use [7].

A randomized control trial (RCT) of COPD patients receiving either once daily 500 mg of ASA or placebo for 12 weeks in addition to their preexisting medication was stopped after an interim analysis was performed as the treatment had no effect on the lung function, measured as forced expiratory volume in 1 s (main outcome),dyspnea (measured by transition dyspnea index (TDI)) or quality of life (St. George’s Respiratory Questionnaire (SGRQ)) [44].

From our point of view, a trial looking at whether antiplatelet therapy might be effective in AECOPD in terms of mortality or acute coronary events instead of lung function would add relevant evidence to adequately manage these patients.

### 5.2. β Blockers

An important matter is the use of β blockers, especially cardio-selective ones in COPD patients as secondary prevention [45]. Beta-blocker therapy has a proven survival benefit in patients with coronary artery disease. Unfortunately, COPD patients with comorbid cardiac disease are less likely to be prescribed β blockers as secondary prevention [46].

Data from the UK national registry of myocardial infarction (Myocardial Ischemia National Audit Project (MINAP) also reported that secondary prevention was less likely to be prescribed in patients with COPD and acute cardiac syndrome. These results were particularly evident for beta blockers (with an odds ratio of 0.25 (95% CI 0.24–0.25) for patients with non-ST elevation myocardial infarctions and 0.26 (95% CI 0.25–0.27) for those with ST-elevation myocardial infarction) [47].

This is probablydue to the ongoing fear of inducing bronchoconstriction. However, a Cochrane systematic review analyzed this issue and described that cardio-selective beta-blockers, given to patients with COPD in the identified studies, did not produce adverse respiratory effects [48].

Some non-randomized studies suggest that beta-blockers have also been associated with reduced exacerbation rates [49] and that mortality is lower in patients taking beta-blockers at the time of an exacerbation than in those who do not [50,51].

A meta-analysis of about fifteen original observational cohort studies revealed that beta-blockers treatment significantly decreased the risk of overall mortality (RR 0.72,95% CI 0.63–0.83) and exacerbation of COPD (RR 0.63,95% CI 0.57–0.71). Indeed, the risk for mortality was more significantly decreased in COPD patient with coronary artery disease (RR 0.64,95% CI 0.54–0.76) [52].

From a clinical stand point, an acute exacerbation of COPD may be a good opportunity to check for a correct diagnosis and treatment of a possible unrecognized CHD.

### 5.3. Statins

Other drugs that have changed prognosis in the cardiovascular setting are statins. The results of retrospective studies suggest that mortality may be lower in patients with COPD who take these drugs than in those who do not [53,54]. One randomized study using simvastatin versus placebo in the prevention of COPD exacerbations did not finddifferences in exacerbation rates, time to first exacerbation, serious adverse events, and mortality [55]. However, this study had a follow up of (±SD) of 641 ± 354 days and we donot know if longer duration of treatment would change the results. Indeed, patients with cardiovascular diseases who may have more impact on treatment were excluded.

Data on previous COPD treatment and acute ischemic heart disease are as follows:

Bronchodilators, principally long-acting muscarinic antagonists (LAMAs) and long-acting beta agonists (LABAs) have long been the mainstay pharmacological treatment of COPD, despite some observational evidence that their use may worsen existing underlying CVD or even increase the risk of developing CVD including cardiac ischemia [56].

However, accumulated evidence from previous clinical trials suggests that inhaled COPD therapies do not pose a significant CVD risk, at least in people free from cardiovascular comorbidities [56].

Lately, some randomized clinical trials on COPD medication including patients with history of heart disease showed more evidence of the cardiovascular safety of LAMA, LABA and inhaled corticosteroids (ICS) [57,58].

In a nested case-control study of more than 280,000 patients with COPD, new use of LABAs or LAMAs was associated with an approximate 1.5-fold increased cardiovascular risk within 30 days of initiation therapy. Although these findings need to be replicated, the results may be explained by the fact that worsening of COPD may have prompted the use of LABAs or LAMAs instead of adequately treat a possible exacerbation of COPD and its increased cardiovascular associated risk [59].

It makes sense that adequately prescribed COPD medications that reduce the risk of AECOPD consequently reduce also its associated increased risk of MI, but this needs to be prospectively verified.

## 6. Conclusions

An early recognition and treatment of ischemic heart diseases in acute exacerbations of COPD may determine prognosis.For this reason, diagnostic techniques for acute IHDshould be accessible for patients with COPD exacerbations. Understanding the complexity of the different possible mechanisms that may contribute to the increased risk of myocardial infarctionin COPD exacerbation, itis important to better delineate possible therapeutic targets in a susceptible population. Cardiac dysfunction during exacerbation represents a research challenge not only in terms of etiology but also concerning treatment strategies.

## Figures and Tables

**Table 1:** Association between acute exacerbations of COPD and acute coronary events: epidemiological studies.

Author	Date	Size	Method	Outcome	Prevalence/Incidence/HR/IRR
Brekke et al. [8]	2008	897	Retrospective study from the Hospital data base	Cardiac Infarction Injury Score (CIIS) to assess the prevalence of prior MI in acute exacerbated COPD patients	229 (25%) patients with ECG signs of previous infarction, only 30% (95% CI 24–36%) had a previous recognized history of MI.
Donaldson et al. [9]	2010	25,857	Retrospective study using UK administrative data	Incidence rates of MI and stroke after exacerbation of COPD	Incidence rates of MI: 1.1 per 100 patient-years Risk of MI 1 to 5 days after AECOPD: IRR 2.27-fold (95% CI 1.1–4.7)
McAllister et al. [10]	2012	242	Prospective case series	Incidence of MI in patients hospitalized with an AECOPD	51% (95% CI 48–58%) develop chest pain; 8.3%; (95% CI 5.1–12.5%) had chest pain and/or serial ECG changes, fulfilling the 2007 Universal Definition of Myocardial Infarction
Kunisaki et al. [11]	2018	16,485	Secondary cohort analysis of the SUMMIT (Study to Understand Mortality and Morbidity) trial	Determine whether AECOPD events are associated with increased risk of subsequent cardiovascular disease	CVD events 30 days following AECOPD: HR 3.8; (95% CI 2.7–5.5) CVD events 30 days following AECOPD with hospitalization: HR 9.9; (95% CI 6.6–14.9).
Rothnie et al. [7]	2018	5696	Retrospective self-controlled case series using UK data database	Quantify the increased risks of MI and ischemic stroke risk associated with both moderate and severe AECOPD	Severe exacerbation-MI risk: IRR 2.58; (95%CI 2.26–2.95) Moderate exacerbation-MI risk: IRR 1.58; (95% CI 1.46–1.71)

AECOPD: Acute exacerbation of COPD; CI: confidence interval; COPD: chronic obstructive pulmonary disease; CVD: cardiovascular disease; ECG: electrocardiogram; HR: hazard ratio; IRR: incident rate ratio; MI: myocardial infarction.
